# Comparative analysis of biological versus chemical synthesis of palladium nanoparticles for catalysis of chromium (VI) reduction

**DOI:** 10.1038/s41598-021-96024-0

**Published:** 2021-08-17

**Authors:** Mpumelelo T. Matsena, Evans M. N. Chirwa

**Affiliations:** Water Utilisation and Environmental Engineering Division, Department of Chemical Engineering, University of Pretoria, Pretoria, 0002 South Africa

**Keywords:** Nanoparticles, Pollution remediation

## Abstract

The discharge of hexavalent chromium [Cr(VI)] from several anthropogenic activities leads to environmental pollution. In this study, we explore a simple yet cost effective method for the synthesis of palladium (Pd) nanoparticles for the treatment of Cr(VI). The presence of elemental Pd [Pd(0)] was confirmed by scanning electron microscope (SEM), electron dispersive spectroscopy and X-ray diffraction (XRD). We show here that the biologically synthesized nanoparticles (Bio-PdNPs) exhibit improved catalytic reduction of Cr(VI) due to their size being smaller and also being highly dispersed as compared to chemically synthesized nanoparticles (Chem-PdNPs). The Langmuir–Hinshelwood mechanism was successfully used to model the kinetics. Using this model, the Bio-PdNPs were shown to perform better than Chem-PdNPs due to the rate constant (k_bio_ = 6.37 mmol s^−1^ m^−2^) and Cr(VI) adsorption constant (K_Cr(VI),bio_ = 3.11 × 10^−2^ L mmol^−1^) of Bio-PdNPs being higher than the rate constant (k_chem_ = 3.83 mmol s^−1^ m^−2^) and Cr(VI) adsorption constant (K_Cr(VI),chem_ = 1.14 × 10^−2^ L mmol^−1^) of Chem-PdNPs. In addition, product inhibition by trivalent chromium [Cr(III)] was high in Chem-PdNPs as indicated by the high adsorption constant of Cr(III) in Chem-PdNPs of K_Cr(III),chem_ = 52.9 L mmol^−1^ as compared to the one for Bio-PdNPs of K_Cr(III),bio_ = 2.76 L mmol^−1^.

## Introduction

Wastewater containing hexavalent chromium [Cr(VI)] is discharged from industrial activities such as electroplating, textile production, leather tanning, paint manufacturing and wood processing to the environment and that leads to heavy metal pollution^1^. The discharge of wastewater bearing Cr(VI) has been associated with deleterious environmental and health effects to plants and mammals (including humans). This is due to the carcinogenicity, mutagenicity and teratogenicity under chronic exposure conditions^1^, and acute under high dose exposure conditions^2^. Several studies have shown that acute exposure to Cr(VI) typically causes indication of skin, bladder, lung and liver cancer^3^ and failure of internal organs^4^. These detrimental effects necessitate the treatment of wastewater containing Cr(VI) before being discharged to the environment.

The most used technique for remediation of Cr(VI) involves chemical or biological reduction of Cr(VI) to the less toxic and less mobile trivalent chromium [Cr(III)], followed by precipitation of Cr(III) and chromium hydroxide [CrOH_3_(s)] at pH 8–9 or through removal of Cr(III) using strong acid cationic resins^5^. It should be noted here that, aside from being easy to precipitate, Cr(III) is a well known essential element for the mammalian nutritional diet. Cr(III) serves as critical cofactor in the pathway for conversion of glucose to energy^6–8^.

The above argument implies that Cr(VI) reduction can be achieved physical-chemically^9^ or biologically^10,11^. Chemically induced Cr(VI) reduction produces several unwanted side effects such utilization of a chemically toxic reactant feedstream and production of toxic sludge^12^. Conversely, biological reduction of Cr(VI) to Cr(III) offers a lower cost alternative process which is much cleaner since no toxic sludge is produced. However, the biological process is much slower which means its application will require longer retention times and therefore much larger reactors than in physical–chemical applications^10^.

Recently, the use of catalytic reduction by nanoparticles as an alternative to physical–chemical and biological Cr(VI) reduction has been receiving considerable attention. Other previously synthesized nanoparticles for catalytic Cr(VI) reduction included silver (Ag)^13^, magnetic iron oxide (Fe_3_O_4_)^14^ and palladium (Pd) nanoparticles^15^. Among those synthesized catalysts, Pd has been explored extensively due to its high selectivity and activity in the oxidation of smaller organic compounds such as formate and lactate^16^. Additionally, the latter method is more favorable as it uses lower cost electron donors and is exempt from Cr(VI) toxicity at higher loading than a purely biological Cr(VI) reduction^15^.

The reduction of Cr(VI) by formate (HCOO^−^) in the presence of Pd nanoparticles occurs via two processes, namely: formate oxidation using Pd nanoparticles, followed by Cr(VI) reduction with the Pd nanoparticles as catalysts. Formate oxidation by Pd nanoparticles occurs via a direct reaction pathway (Eqs. (1)–(3))^17^:

1 $${\text{HCOO}}_{{\rm ad}}^{ - } \to {\text{H}}_{{\rm ad}} + {\text{COO}}_{{\rm ad}}^{ - }$$

2 $${\text{H}}_{{\rm ad}} + {\text{OH}}^{ - } \to {\text{H}}_{2} {\text{O}} + {\text{e}}^{ - }$$

3 $${\text{COO}}_{{\rm ad}}^{ - } \to {\text{CO}}_{2} + {\text{e}}^{ - }$$

This pathway is important since it avoids carbon monoxide (CO) poisoning during formate oxidation on Pd nanoparticles^18,19^. Each of the reactions of the desorption of hydrogen (Eq. (2)) and oxidation of adsorbed COO^−^ (Eq. (3)) contribute one electron charge transfer. Then Cr(VI) reduction occurs via Eq. (4) and is facilitated by the electrons generated from formate oxidation by Pd nanoparticles:

4 $${\text{Cr}}_{2} {\text{O}}_{7}^{2 - } + 14{\text{H}}^{ + } + 6{\text{e}}^{ - } \leftrightarrow 2{\text{Cr}}^{3 + } + 7{\text{H}}_{2} {\text{O}}$$

The standard reduction potential of Cr(VI) is 1.33 V^20^. Additionally, when formate is used as an electron donor (− 0.43 V)^21^, catalytic Cr(VI) reduction using Pd nanoparticles is theoretically possible due to a positive overall standard reduction potential (E°), where E° = 1.76 V. Based on this information, this implies that the treatment of Cr(VI) using the catalytic reduction process through Pd nanoparticles and formate is theoretically feasible and can be achieved with minimum energy losses.

One way which Pd nanoparticles can be synthesized is by using divalent palladium [Pd(II)] in the presence of formate to form chemically synthesized palladium nanoparticles (Chem-PdNPs) as reported by Bunge et al.^22^ and Deplanche et al.^23^. The other method is to biologically synthesize palladium nanoparticles (Bio-PdNPs) using Pd(II) reducing bacteria such as *Shewanella oneidensis* SR-1^24^, *Desulfovibrio desulfuricans*^25^, and/or *Citrobacter* sp.^26^. The advantage of synthesizing Bio-PdNPs using formate is that the process avoids the use of harsh chemical agents. Evidently, the synthesis of Bio-PdNPs using the microbial method offers several advantages in that it occurs under gentle conditions and offers good biocompatibility^24,27^.

Herein, the main objective of this paper was to evaluate both synthesis methods and characterize the formed Pd nanoparticles and perform a comparative analysis upon the performance of nanoparticles emanating from the two protocols. Both methods, if proved viable, will provide a potential for the development of a simple yet cost-effective process for the catalytic reduction of Cr(VI).

## Materials and methods

### Chem-PdNPs and Bio-PdNPs synthesis

Chem-PdNPs were synthesized in Basal mineral medium (BMM) at desired Pd(II) and 5 g L^−1^ sodium formate in 100 mL serum bottles without using microbial culture. To adjust the temperature during Chem-PdNPs synthesis, a heating plate was used. Pd(II) stock solution (1 g L^−1^) was prepared by dissolving 2.48 g of 99% pure Pd(NH_3_)_4_Cl_2_·H_2_O (analytical grade) in 1 L deionized water. It was used throughout the experiments to serve as Pd(II) source. *Citrobacter* sp. which was isolated by Matsena et al.^26^ capable of Bio-PdNPs production was first cultured anaerobically in LB medium for 24 h at 28 °C. The microbial culture was then collected and the production of Bio-PdNPs proceeded anaerobically in the BMM^26^ at desired Pd(II) concentration and 5 g L^−1^ sodium formate in 100 mL serum bottles according to the method previously described by Wang et al.^15^.

### Comparative Cr(VI) reduction using Chem-PdNPs and Bio-PdNPs

Since *Citrobacter* sp. used to synthesize Bio-PdNPs is a mesophile^26^, the bacterial cells were heat killed by autoclaving at 121 °C at 115 kg cm^−2^ for 15 min before use of the Bio-PdNPs to ensure that they do not contribute to Cr(VI) reduction. The Chem-PdNPs and Bio-PdNPs suspensions were then collected by centrifuging at 6000 rpm for 15 min and resuspended in 10 mL BMM to be tested for catalytic activity in Cr(VI) catalytic reduction experiments. Cr(VI) stock solution (1 g L^−1^) was prepared by dissolving 2.83 g of 99% K_2_Cr_2_O_7_ (analytical grade) in 1 L deionized water. It was used throughout the experiments to serve as Cr(VI) source. Catalytic Cr(VI) reduction was then carried out at a room with a temperature of 30 ± 2 °C without any continuous shaking in 100 mL serum bottles at a desired Cr(VI) concentration and 5 g L^−1^ sodium formate using the synthesized Pd nanoparticles. It should be noted that prior to the experiments, the serum bottles were purged with nitrogen gas to remove dissolved oxygen.

### Cr(VI) concentration analysis

Cr(VI) was measured in water samples by a UV/Vis spectrophotometer (WPA, Light Wave II, Labotech, South Africa) using the method described earlier by Chirwa and Wang^28^. The measurements were carried out at a wavelength of 540 nm (10 mm light path) after acidification of 0.2 mL samples with 2 mL of 1 M H_2_SO_4_ and dilution with distilled water to 10 mL, followed by reaction with 0.2 mL of a 15% solution of 1,5-diphenyl carbazide to produce a purple colour^29^. The removal percentage of Cr(VI) was calculated as removal % = [(initial Cr(VI) − final Cr(VI))/initial Cr(VI)] × 100.

### Total Cr and Cr(III) concentration analysis

The determination of total chromium (Cr) was conducted using a AAnalyst 400, S/N 201S8070301 atomic absorption spectrometer fitted with a Model 510 auto sampler. The analysis was conducted using a 3 mA chromium hollow cathode lamp at a wavelength of 359.9 nm. Cr(III) concentration in the solution was determined as a difference between total Cr and Cr(VI) since total Cr measures the sum of aqueous Cr(VI) and Cr(III) concentrations^30^.

### Pd(II) concentration analysis

Pd(II) concentration was determined by atomic absorption spectrometry (AAS) using an AAnalyst 400, S/N 201S8070301 Auto sampler Model 510, with a Parkin-Elmer Lumina Pd hollow cathode lamp (Perkin-Elmer, Palo Alto, California, USA) at a wavelength of 244.79 nm.

### Pd nanoparticles characterization

#### Morphology analysis

Morphology analysis of Chem-PdNPs and Bio-PdNPs was conducted by scanning electron microscopy (SEM). The carrier samples were allowed to air dry and pieces on each sample were removed and mounted with adhesive carbon tape on aluminium stubs in the upright position^31^. These were viewed in a Zeiss Ultra Plus field emission scanning electron microscope (FE-SEM) (Zeiss, Germany) at 2 kV.

#### Elemental composition analysis

To determine the elemental composition of Chem-PdNPs and Bio-PdNPs, electron dispersive spectroscopy (EDS) was used. The carrier samples were allowed to air dry and pieces on each sample were removed and mounted with adhesive carbon tape on aluminium stubs in the upright position. The EDS analysis was performed using the (AZtecEnergy) software (Oxford Instruments, UK) linked to an Oxford detector (Oxford Instruments, UK) with an 80 mm^2^ detection window.

#### XRD analysis

For phase identification and determining information on the unit dimensions, X-ray Powder Diffraction was used. XRD was conducted using a PANanalytical X’Pert Pro Powder diffractometer in θ–θ configuration with an X’Celerator detector and variable divergence and fixed receiving slits with Fe filtered Co-Kα radiation (λ = 1.789 Å). The mineralogy was determined by selecting the best fitting pattern from the ICSD database to the measured diffraction pattern using X’Pert Highscore plus software (version 3.0). The crystallite size was determined using the Scherrer equation, *D* = *kλ*/(*βcosθ*), where k is the shape factor constant (0.9), λ is the X-ray wavelength (0.179 nm), β is the full width at half maximum intensity in radians, and θ is the bragg angle in degrees.

## Results and discussion

### Optimization of Chem-PdNP and Bio-PdNP synthesis

#### Effect of pH on Chem-PdNPs and Bio-PdNPs formation

**Figure 1:**
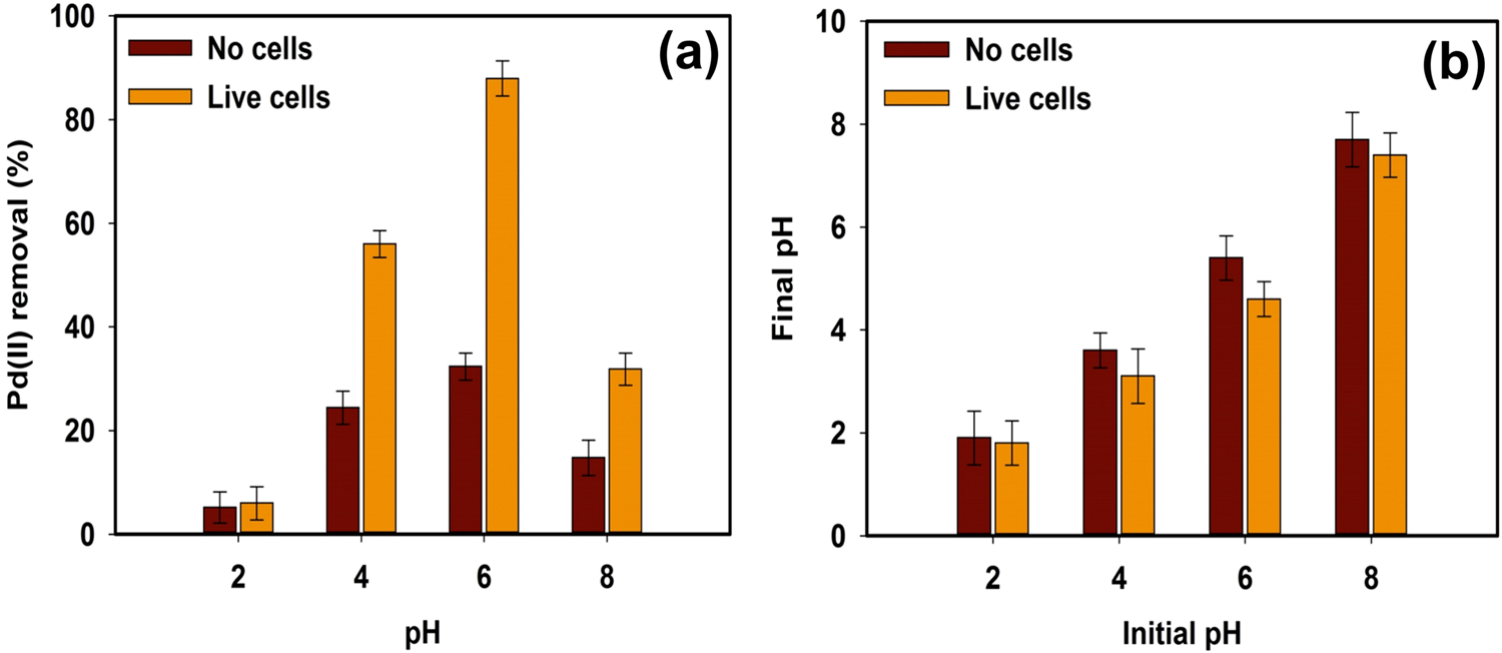
The effect of initial pH on (**a**) Pd(II) removal from the aqueous phase and (**b**) final pH using no microbial cells and live microbial cells.

The method detailed in this study for the synthesis of Chem-PdNPs and Bio-PdNPs is simple and requires only Pd(II) and formate as the main chemical agents. However, optimal synthesis parameters need to be obtained before the nanoparticles can be produced for the catalytic reduction of Cr(VI). The results of pH optimization are shown in Fig. 1. The synthesis of the nanoparticles for experiments with and without microbial cells at both acidic (pH 2) and basic (pH 8) conditions resulted in low Pd(II) removal (Fig. 1a). The pH value of 6 led to the highest Pd(II) removal of 87.9% and 32.2% for each experiment with and without microbial cells, respectively.

The reason acidic (pH 2) conditions led to low removal was because Pd(II) is predominant in the form of palladium tetra-ammine complex (Pd(NH_3_)_4_^2+^) between pH 5–13.6, and below pH 5, it is predominant in the form of tetrachloropalladate complex ([PdCl_4_]^2−^)^32^, which means that [PdCl_4_]^2−^ specie was not easily reduced by formate. On the other hand, the decrease in Pd(II) removal at basic (pH 8) conditions was related to an increased precipitation due to the interaction between Pd(II) and hydroxyl ions in the aqueous solution forming insoluble metal precipitates^26^.

The reason the experiment with microbial cells led to the highest Pd(II) removal as compared to the experiment without microbial cells was because *Citrobacter* sp. cells improved the ability of Pd(II) to biosorb on the microbial cells and enhance the enzymatic Pd(II) reduction^26^. This was attributed to the fact that most bacteria start to achieve a negative electrophoretic mobility (EPM) between pH 3–4 when the pH is increased^33,34^. This negative EPM indicates that the microbial cells have a negative surface charge. Additionally, the use of Pd(II) as Pd(NH_3_)_4_^2+^ facilitated the attraction of this cationic palladium species towards the negatively charged *Citrobacter* sp. cell surfaces before the Pd(II) reduction and biocrystallization occurred on the cell surfaces. In this way, the microbial cells functioned as biocatalysts to the chemical reduction of Pd(II) by formate.

The final pH of the microbial cells experiments was lower than the final pH of the experiments with no microbial cells (Fig. 1b). This observation can be explained when we consider the half redox reactions^21,35^:

5 $$3{\text{Pd}}\left( {{\text{NH}}_{3} } \right)_{4}^{2 + } + 6{\text{e}}^{ - } \leftrightarrow 3{\text{Pd}} + 12{\text{NH}}_{3} \; {\text{E}}^{0} = 0.0{\text{V}}$$

6 $$3{\text{HCOO}}^{ - } \leftrightarrow 3{\text{CO}}_{2} + 3{\text{H}}^{ + } + 6{\text{e}}^{ - } \; {\text{E}}^{0} = - 0.43{\text{V}}$$

7 $$3{\text{Pd}}\left( {{\text{NH}}_{3} } \right)_{4}^{2 + } + 3{\text{HCOO}}^{ - } \leftrightarrow 3{\text{Pd}} + 12{\text{NH}}_{3} + 3{\text{CO}}_{2} + 3{\text{H}}^{ + } \; {\text{E}}^{0} = 0.43{\text{V}}$$

As it can be seen from the net redox reaction in Eq. (7), the oxidation of formate by Pd(II) produced hydrogen ions (H^+^) which reduced the pH. Therefore, higher Pd(II) removal rates are expected to produce lower final pH values in batch processes.

#### Effect of Temperature on Chem-PdNPs and Bio-PdNPs formation

**Figure 2:**
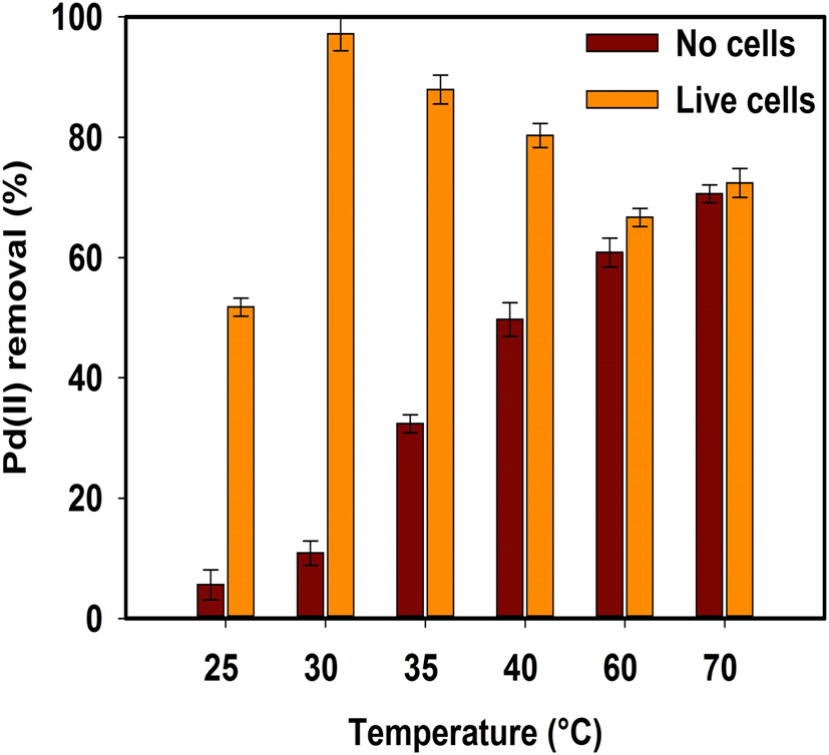
Temperature effect on the Pd(II) removal during Pd nanoparticles synthesis using no microbial cells and live microbial cells.

The influence of temperature on the formation of both Chem-PdNPs and Bio-PdNPs was evaluated and the results are shown in Fig. 2.

It can be observed that when the temperature was increased from 25 to 70 °C, the Pd(II) removal from solution in systems with no microbial cells increased from 5.6 to 70.6%. Notably, the experiment with live microbial cells achieved the highest removal of Pd(II) of 97.2% at 30 °C.

Normally the reaction rate for the chemical synthesis of nanoparticles increases with an increase in reactive temperature. This is due to the temperature having the ability to increase the rate of crystal growth of the nanoparticles^36^. This in turn increases the formation of nucleation sites for Pd nanoparticles deposition and promotes crystal growth which is self-sustaining due to autocatalytic reduction of more Pd(II)^37^. However, for the biological synthesis, this is different due to the enzyme activity of microbial cells being sensitive to the change in temperature.

Microbial cells can be classified based on the temperature in which they achieve optimum growth. Psychrophiles grow best between − 5 and 20 °C, mesophiles temperatures are between 20 and 45 °C, and thermophiles temperatures are above 45 °C. Since the microbial cells used in this study which are *Citrobacter* sp. are mesophilic^26^, the highest Pd(II) removal was achieved at temperatures between 20 and 45 °C. It should be noted that the decrease in Pd(II) removal by the use of *Citrobacter* sp. after 30 °C was caused by the denaturation of proteins since mesophiles are not able to survive at thermophilic temperatures^38,39^.

The reason biological synthesis is better than chemical synthesis at low temperatures is due to the enhanced enzyme activity of the microbial cells at low temperatures and the microbial cells themselves serving as nucleation sites which improves Bio-PdNPs formation. At high temperatures, an interesting observation is made where the live microbial cells Pd(II) removal is approaching the Pd(II) removal of no microbial cells. This is attributed to the fact that mesophilic microorganisms are not able to survive thermophilic temperatures as previously mentioned. Therefore, the results at 60 °C and 70 °C in the experiments with microbial cells are a reflection of the chemical synthesis of Pd nanoparticles since there is no enzyme activity due to dead cells.

#### Effect of Pd(II) concentration on Chem-PdNPs and Bio-PdNPs formation

**Figure 3:**
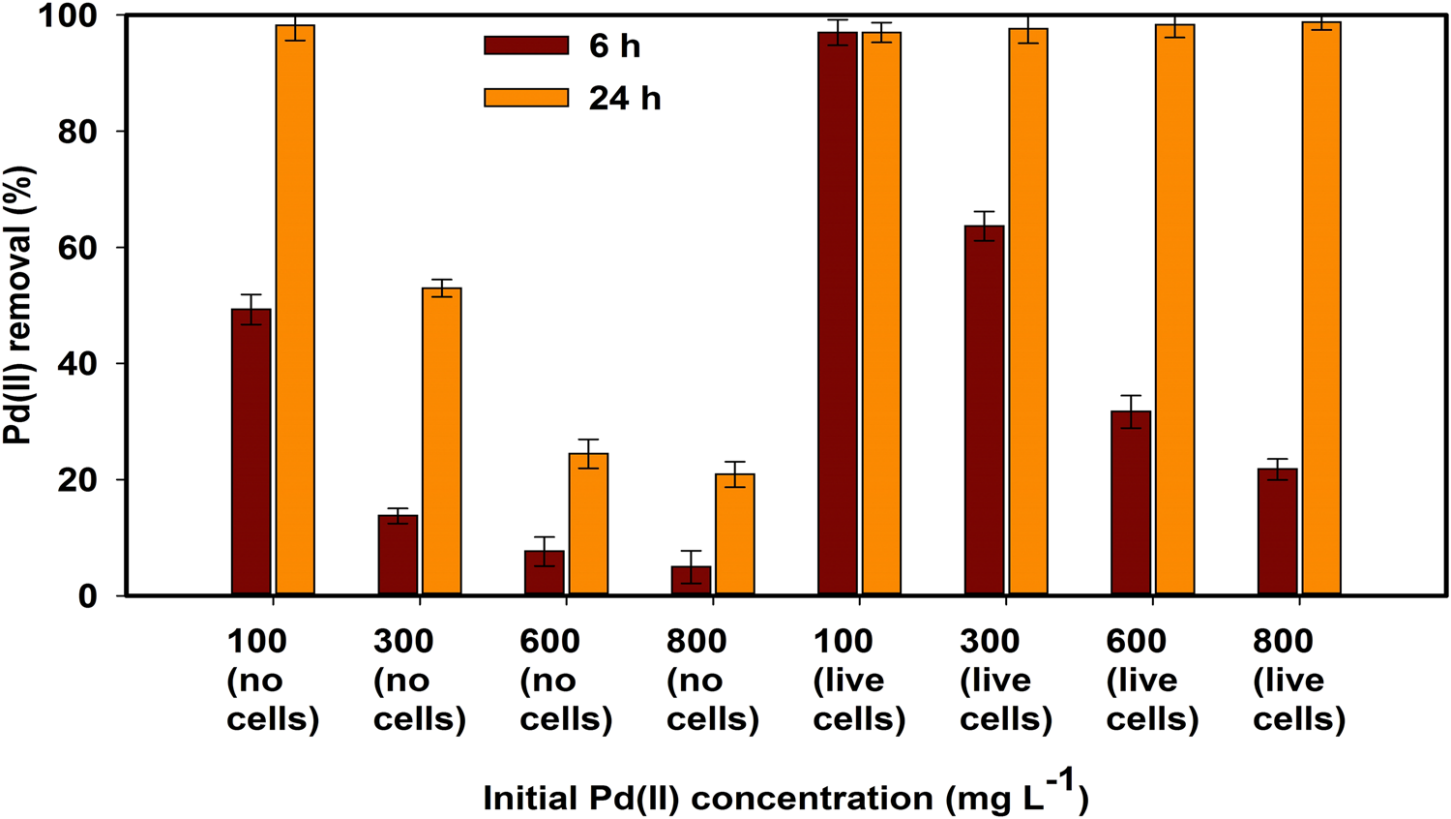
Initial Pd(II) concentration effect on the Pd(II) removal measured at 6 h and 24 h during Pd nanoparticles synthesis using no microbial cells and live microbial cells.

The initial concentration of Pd(II) affects the time it takes to achieve complete Pd(II) removal as shown in Fig. 3.

Figure 3 shows that when the initial concentration of Pd(II) is increased from 100 to 800 mg L^−1^, Pd(II) removal decreases at 6 h for the experiment with and without microbial cells. It is only after 24 h that the experiment with microbial cells achieves complete removal no matter the initial concentration as compared to the experiment without the microbial cells. This is an advantage as the biological removal of Pd(II) provides the capability to operate at a wide range of initial Pd(II) concentrations which allow for the complete removal in 24 h.

The microbial cells have been found to improve nucleation sites for the development of nanoparticles which in turn improves the crystal growth and autocatalytic reduction of Pd(II)^37^. In addition, *Citrobacter* sp. can biosorb and enzymatically reduce Pd(II)^26^. This in turn improves the rate at which the nanoparticles are being formed since biosorption involves the reversible and rapid binding of ions onto the functional groups on the surface of microbial cells. From Fig. 3, it can be seen that the presence of microbial cells improves the rate of removal of Pd(II) as compared to the experiment without microbial cells, which shows that Pd nanoparticle formation was faster in the presence of living cells and indicates that Pd(II) reduction was metabolically linked.

#### Visual observation of Chem-PdNPs and Bio-PdNPs

Supplementary Fig. S1 online shows the visual observation of the Chem-PdNPs and Bio-PdNPs. The Chem-PdNPs form a chunk of nanoparticles at the bottom of the serum bottle which are aggregated and the Bio-PdNPs form a black solution containing highly dispersed nanoparticles.

It has been shown that the carbonyl groups of the enzymes and proteins secreted by microbial cells can help stabilize biologically synthesized nanoparticles^40^. In addition, some functional groups such as –OH, –SH, –COOH and –NH_2_ of the proteins secreted by the microbial cells play an important role in the reduction of metals and the subsequent stabilization of the synthesized nanoparticles^41^. For example, a study done by Li et al.^42^ showed that protein extracts of *Deinococcus radiodurans* can be used to stabilize gold nanoparticles*.* Therefore, the reason the Bio-PdNPs formed less aggregated nanoparticles in this study might have been attributed to the secretion of stabilizing agents by the *Citrobacter* sp. cells.

Various stabilizing agents can be used which include surfactants, natural polymers, bi-functional organic compounds, and oligomers^43^. However, such stabilizers result in very high financial inputs and toxic final effluent after the expiry of the nanoparticles. Since this study shows that microbial cells can serve as stabilizing agents due to the Bio-PdNPs being less aggregated, it means that the microbial cells can be used as a substitute to the costly stabilizing agents that are utilized during the synthesis of nanoparticles. This is in agreement with observations made by Abd Razak and Shamsuddin^44^ where it was shown that nanoparticles can be biologically stabilized.

### Characterization of Chem-PdNPs and Bio-PdNPs

**Figure 4:**
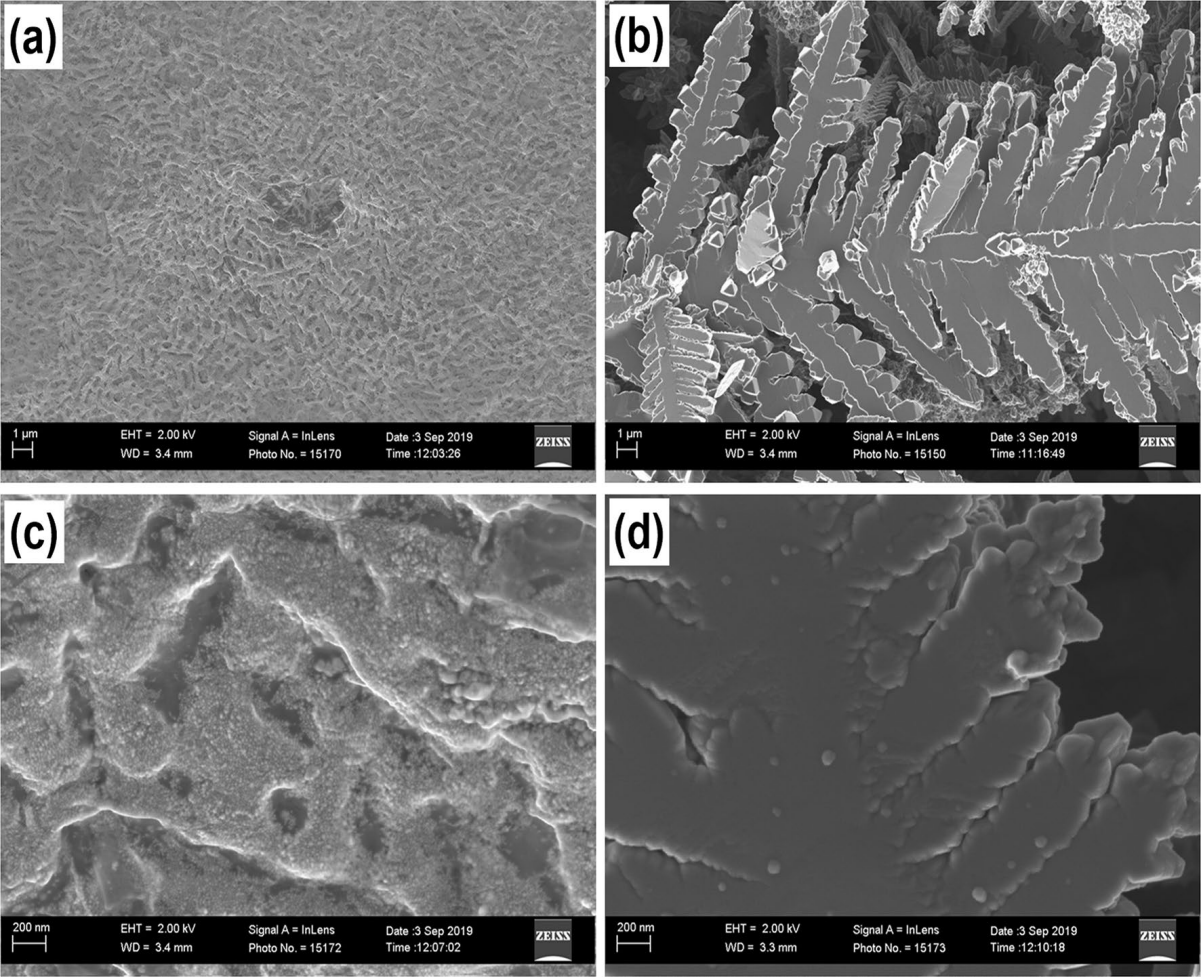
SEM morphology observations at 1 mµ magnification for (**a**) Bio-PdNPs and (**b**) Chem-PdNPs; and at 200 nm magnification for (**c**) Bio-PdNPs and (**d**) Chem-PdNPs.

Given that we are synthesizing Pd nanoparticles, it was important to validate if elemental palladium [Pd(0)] was formed through the chemical and biological synthesis methods. This was done by conducting characterization studies on the formed Pd product. The optimized synthesis parameters are shown in Supplementary Table S1 online for the fabrication of Pd nanoparticles to be characterized. The morphology analysis of the formed Pd nanoparticles (Fig. 4) was evaluated using the scanning electron microscopy (SEM).

During the 1 mµ magnification of the Bio-PdNPs shown in Fig. 4a, the Pd nanoparticles attached on the surface of the *Citrobacter* sp. cells did not show any apparent shape or deposition of the nanoparticles. However, the Chem-PdNPs under 1 mµ magnification already showed the development of a flower-shape with a number of petals as shown in Fig. 4b. This gives an indication that the Bio-PdNPs are smaller in size as compared to the Chem-PdNPs. When the magnification was increased to 200 nm for the Bio-PdNPs, the micrograph showed a roughened surface with many random and irregular arranged protrusions of the nanoparticles (Fig. 4c). In addition, the 200 nm magnification for the Chem-PdNPs showed how each petal had several smaller protrusions of nanoparticles (Fig. 4d).

**Figure 5:**
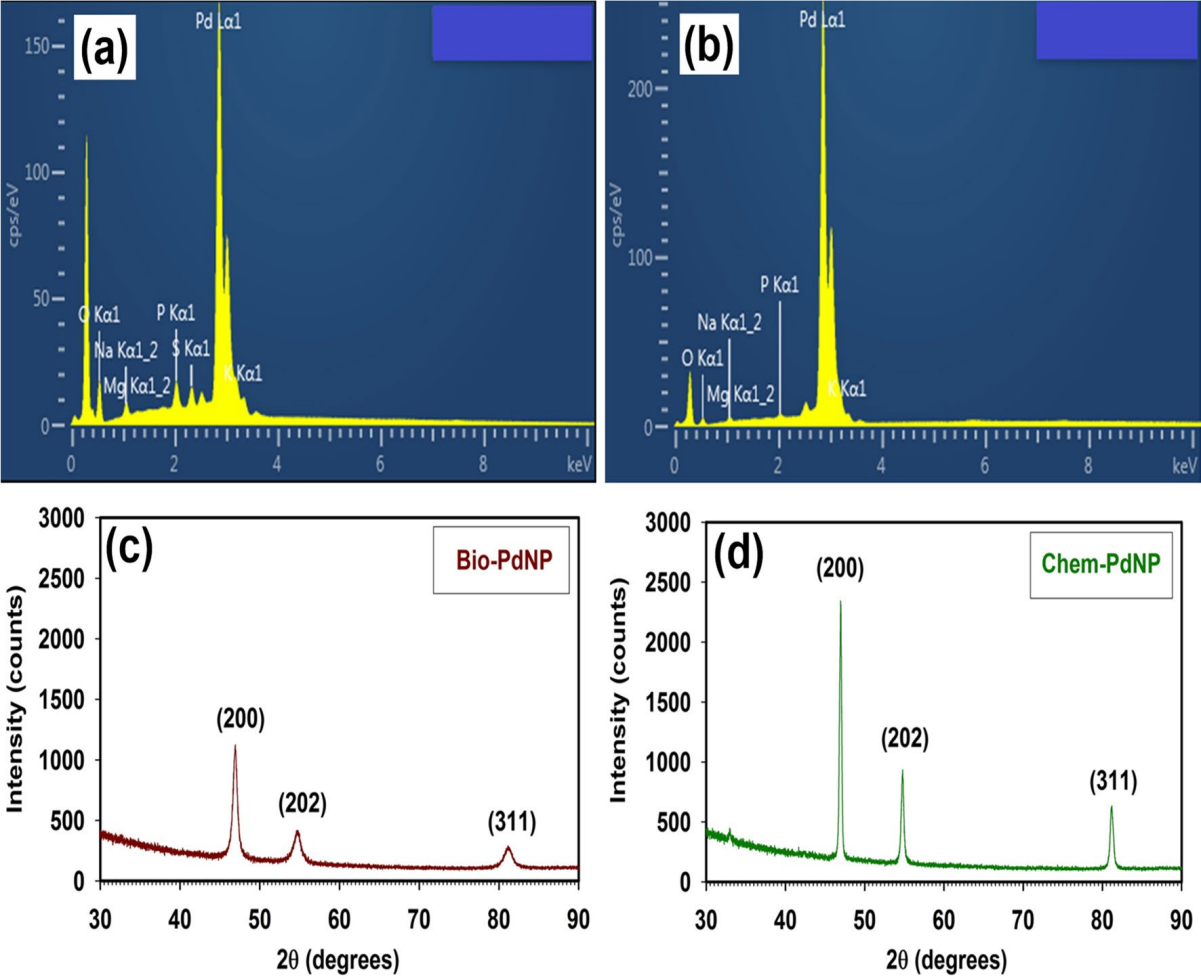
Elemental composition analysis by EDS of (**a**) Bio-PdNPs and (**b**) Chem-PdNPs; and XRD patterns of (**c**) Bio-PdNPs and (**d**) Chem-PdNPs.

To confirm if the resultant protrusion in Bio-PdNPs and Chem-PdNPs were actually Pd, the electron dispersive spectroscopy (EDS) was used to confirm the presence of Pd in the resultant solids with the Pd peaks being detected as shown in Fig. 5a and b. Further characterization of the solids was conducted using X-ray diffraction (XRD) in order to identify the specific crystal phases present in the solids. The obtained spectra for Bio-PdNPs in Fig. 5c and Chem-PdNPs in Fig. 5d confirmed the presence of elemental Pd(0) mixed with palladium (II) oxide (PdO). The peaks corresponding to 2θ values of 46.54° and 82.18° were attributed to Pd(0) and matched similar studies by Hazarika, et al.^27^ (JCPDS card No. 001–1201). They also reported the presence of a PdO peak at a 2θ value of 55.86°, which is similar to the peak with coordinates of (202) shown in the present results (JCPDS card No. 002–1432)^27^.

To ascertain if the formed Pd(0) are nanoparticles, the Scherrer equation was used to calculate the crystallite size using the results obtained in Fig. 5c and d. The sizes of Pd(0) at 2θ values of 46.54° and 82.18° were calculated as 17.6 nm and 25.84 nm for Bio-PdNPs, respectively. In addition, the sizes of Pd(0) at 2θ values of 46.54° and 82.18° were calculated as 39.78 nm and 91.5 nm for Chem-PdNPs, respectively.

The reason the size of the Bio-PdNPs were smaller than the Chem-PdNPs is related to the fact that the presence of the microbial cells increases the nucleation sites for Pd nanoparticles deposition and promotes crystal growth which is self-sustaining due to autocatalytic reduction of more Pd(II)^37^. This then leads to smaller nanoparticle sizes since it has been shown in previous studies that the nucleation rate plays an important role in the particle sizes that are formed. The higher nucleation rate, the lower the nanoparticle sizes^45^.

### Cr(VI) reduction using Chem-PdNPs and Bio-PdNPs

**Figure 6:**
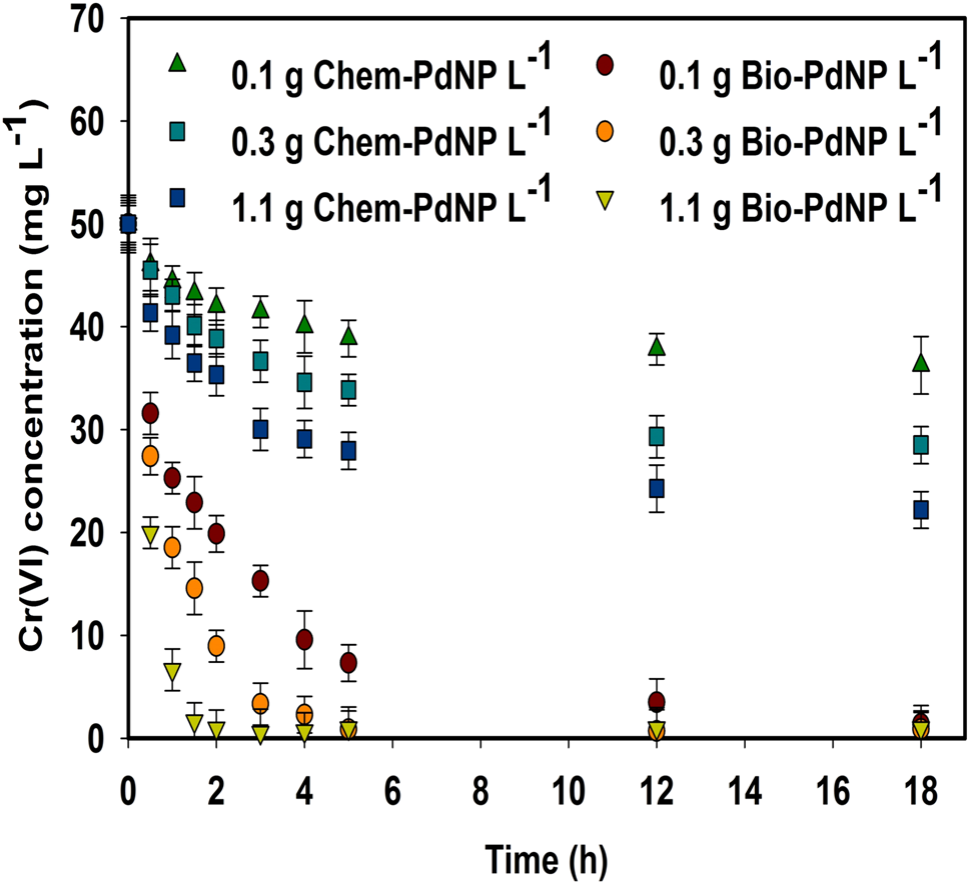
Cr(VI) reduction using Chem-PdNPs and Bio-PdNPs of different catalyst concentrations.

The catalytic reduction of Cr(VI) was studied under different catalyst concentrations of Chem-PdNPs and Bio-PdNPs as shown in Fig. 6. It should be noted that *Citrobacter* sp. used to synthesize Bio-PdNPs is a mesophile^26^ and since the bacterial cells were heat killed by autoclaving at 121 °C at 115 kg cm^−2^ for 15 min before use of the Bio-PdNPs, they then did not contribute to Cr(VI) reduction.

The usage of Pd nanoparticles which were synthesized via the chemical synthesis method performed lower than the ones which were fabricated biologically. When considering only the Chem-PdNPs, the highest Cr(VI) removal rate of 55.6% was achieved when using the catalyst concentration of 1.1 g Chem-PdNP L^−1^. On the other hand, complete removal of Cr(VI) was achieved when considering the Bio-PdNPs of all catalyst concentrations. The performance and the rate of Cr(VI) removal was enhanced when increasing the catalyst concentration of both Chem-PdNPs and Bio-PdNPs.

The process of formate oxidation is enhanced by the synthesized Pd nanoparticles. The generated electrons as per Eqs. (2) and (3) are used to reduce Cr(VI) as per Eq. (4). This process is known as catalytic reduction of Cr(VI) using formate and Pd nanoparticles and it is demonstrated in this study. The reason the Bio-PdNPs performed better than the Chem-PdNPs is attributed to the Bio-PdNPs being highly dispersed as compared to the Chem-PdNPs. This was confirmed by the visual representation of Bio-PdNPs as compared to the Chem-PdNPs. In addition, the Bio-PdNPs remained as small sized nanoparticles while the Chem-PdNPs aggregated into larger nanoparticles as confirmed by the XRD results and the Scherrer equation.

The difference in the nanoparticles dispersion and size has been shown to affect their catalytic performance. This is because in principle, when the dispersion of the nanoparticles is increased, the particle size decreases and leads to an increase in active surface area and the number of active sites^46^. This is called a positive particle size effect^47^. Previous studies which have also observed this include a study by Jawale et al.^48^ which observed gold nanoparticles catalytic activity on organic transformations to increase with a decrease in size, and a study by Bokhimi et al.^49^ which led to an increase in the conversion of CO during its oxidation with a decrease in gold nanoparticle size from 6 to 3 nm. However, large particle sizes can be more active than the small particles. This can result to a decrease in catalytic activity with a decrease in particle size. This is called a negative particle size effect^50^.

It should be noted that although both chemical and biological methods are simple and cost-effective, the catalytic reduction of Cr(VI) using the biological synthesis method proved to be more favorable. This was attributed to the high dispersion of nanoparticles in the biologically mediated synthesis system.

### Kinetics for catalytic Cr(VI) reduction using Chem-PdNPs and Bio-PdNPs

#### Kinetic model

Various mechanisms can be used to describe the heterogeneous catalytic reduction process. These include the Eley–Rideal mechanism and the Langmuir–Hinshelwood mechanism. The Eley–Rideal mechanism considers a case where only one of the reactants is adsorbed on the surface of the catalyst and then reacts with other reactants. On the other hand, the Langmuir–Hinshelwood mechanism is used to describe the bimolecular reactions on the catalysts surfaces and considers a case where two reactants get adsorbed on the surface of the catalyst and then react together afterwards.

Various studies have modelled the heterogeneous catalytic reduction which uses nanoparticles as catalysts and have found the Langmuir–Hinshelwood mechanism to perform better. In addition, Yang et al.^51^ found that the catalytic reduction of Cr(VI) using palladium nanoparticles and formic acid as a substrate can be modelled using the Langmuir–Hinshelwood mechanism. It should be noted that some studies reported the use of Michaelis–Menten model, however, Gu et al.^52^ reported that although the model can be justified for biochemical reactions, its general validity for catalytic reactions using nanoparticles can be doubted.

**Figure 7:**
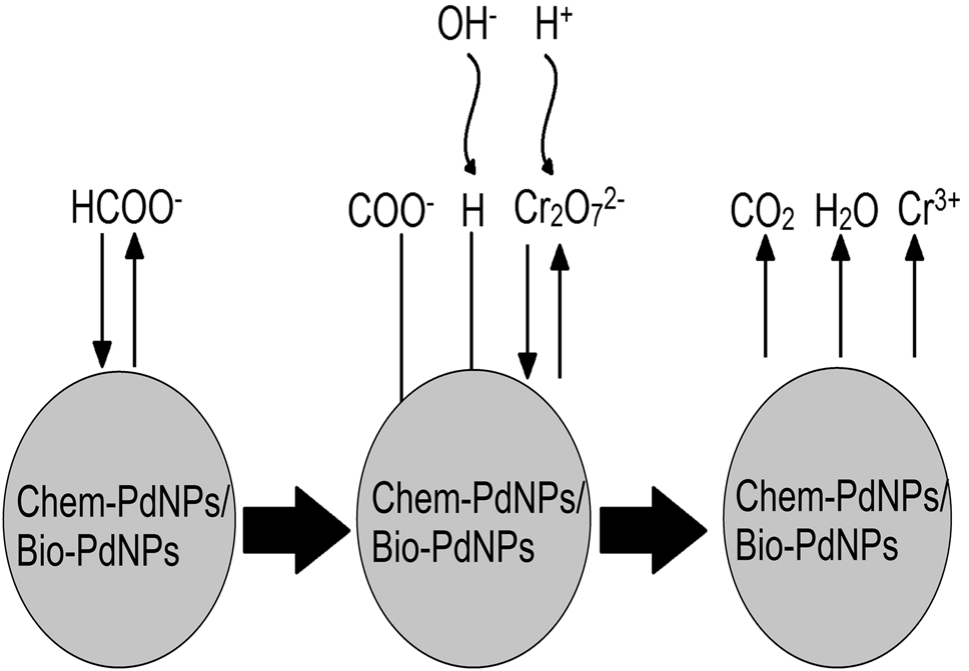
Schematic for the reduction of Cr(VI) using the Langmuir–Hinshelwood kinetics. The surface of the Chem-PdNPs and Bio-PdNPs is where the reaction is taking place. Formate (HCOO^−^) is adsorbed on the surface and forms adsorbed H_(ads)_ and COO^−^_(ads)_ intermediates according to Eq. (1). The intermediates donate electrons according to Eqs. (2) and (3) to the adsorbed dichromate (Cr_2_O_7_^2−^) in the presence of hydrogen ions and hydroxide ions. Upon acceptance of electrons, Cr_2_O_7_^2−^ reduces to Cr^3+^ according to Eq. (4) and the products desorb from the surface to start a new catalytic cycle.

Therefore, in this study, Langmuir–Hinshelwood mechanism was used to model Cr(VI) reduction using Chem-PdNPs and Bio-PdNPs. Figure 7 shows the schematic for the Langmuir–Hinshelwood mechanism used in this study for the model. This proposed model is consistent with the studies done by Wang et al.^17^ for formate oxidation and by Yang et al.^51^ for catalytic Cr(VI) reduction using palladium nanoparticles.

Model assumptions include: (1) the reaction is taking place on the surface of Chem-PdNPs and Bio-PdNPs, (2) formate and dichromate are the two main reactants which adsorb on the neighbouring sites, (3) the formation of intermediates including adsorption and desorption is assumed to be fast, and therefore, the rate-determining step is given by the reaction of formate and dichromate, (4) the rate-determining step is irreversible, (5) formate is present in excess, and (6) formation of Cr(III) inhibits the catalytic reduction of Cr(VI).

In order to model the catalytic Cr(VI) reduction, we consider the Langmuir isotherm given by Eq. (8):

8 $$\uptheta _{{\rm i}} = \frac{{{\text{K}}_{{\rm i}} {\text{C}}_{{\rm i}} }}{{1 + \mathop \sum \nolimits_{{{{\rm j}} = 1}}^{{\text{N}}} {\text{K}}_{{{\rm j}}} {\text{C}}_{{\rm i}} }}$$

where θ_i_ is the surface coverage of component i, K_i_ represents the adsorption constant of component i, C_i_ is the concentration of component i, and N is the number of components. Since the main assumption for the model is the Langmuir–Hinshelwood mechanism, Eq. (9) is used as an expression for the reaction rate equation^52^:

9 $$\frac{{{\text{dC}}_{{{{\rm Cr}}\left( {{\text{VI}}} \right)}} }}{{{\text{dt}}}} = - {\text{kS}}\uptheta _{{{{\rm Cr}}\left( {{\text{VI}}} \right)}}\uptheta _{{{{\rm HCOO}}^{ - } }}$$

where ${\text{C}}_{{{{\rm Cr}}\left( {{\text{VI}}} \right)}}$ is the concentration of Cr(VI), k is the molar rate per square meter of the catalyst, S is the total surface area of the nanoparticles per reaction volume, and $\uptheta _{{{{\rm Cr}}\left( {{\text{VI}}} \right)}}$ and $\uptheta _{{{\text{HCOO}}^{ - } }}$ are the surface coverage of Cr(VI) and formate respectively. By expressing the surface coverage of each component in Eq. (9) based on Eq. (8), the resulting expression is shown in Eq. (10):

10 $$\frac{{{\text{dC}}_{{{{\rm Cr}}\left( {{\text{VI}}} \right)}} }}{{{\text{dt}}}} = - \frac{{{\text{kSK}}_{{{{\rm Cr}}\left( {{\text{VI}}} \right)}} {\text{C}}_{{{{\rm Cr}}\left( {{\text{VI}}} \right)}} {\text{K}}_{{{\text{HCOO}}^{ - } }} {\text{C}}_{{{\text{HCOO}}^{ - } ,{\text{o}}}} }}{{\left( {1 + {\text{K}}_{{{{\rm Cr}}\left( {{\text{VI}}} \right)}} {\text{C}}_{{{{\rm Cr}}\left( {{\text{VI}}} \right)}} + {\text{K}}_{{{\text{HCOO}}^{ - } }} {\text{C}}_{{{\text{HCOO}}^{ - } ,{\text{o}}}} + {\text{K}}_{{{{\rm Cr}}\left( {{\text{III}}} \right)}} {\text{C}}_{{{{\rm Cr}}\left( {{\text{III}}} \right)}} } \right)^{2} }}$$

where ${\text{K}}_{{{{\rm Cr}}\left( {{\text{VI}}} \right)}}$, ${\text{K}}_{{{\text{HCOO}}^{ - } }}$, ${\text{K}}_{{{{\rm Cr}}\left( {{\text{III}}} \right)}}$ are the adsorption constants of Cr(VI), formate, and Cr(III) respectively, ${\text{C}}_{{{\text{HCOO}}^{ - } ,{\text{o}}}}$ is the initial concentration of formate and ${\text{C}}_{{{{\rm Cr}}\left( {{\text{III}}} \right)}}$ is the concentration of Cr(III). Equation (10) assumes that formate is in excess and its concentration change is negligible, and that there is inhibition in the catalytic reduction of Cr(VI) due to the formation of Cr(III). The product inhibition term was suggested by Gu et al.^52^.

The concentration of Cr(III) can be expressed in terms of Eq. (11):

11 $${\text{C}}_{{{{\rm Cr}}\left( {{\text{III}}} \right)}} = {\text{C}}_{{{{\rm Cr}}\left( {{\text{VI}}} \right),{\text{o}}}} - {\text{C}}_{{{{\rm Cr}}\left( {{\text{VI}}} \right)}}$$

where ${\text{C}}_{{{{\rm Cr}}\left( {{\text{VI}}} \right),{\text{o}}}}$ is the initial concentration of Cr(VI). To calculate S, the total Pd surface area (A) has to be considered and it can be calculated using Eq. (12)^51^:

12 $${\text{A}} = \left( {\frac{{\text{m}}}{{\uprho \cdot 4/3\uppi {\text{R}}^{3} }}} \right)4\uppi {\text{R}}^{2}$$

where m is the mass of Pd catalyst, ρ is the density of the metallic Pd (11.8 × 10^3^ kg m^−3^)^53^, and R is the radius of the Pd nanoparticles and is calculated from average of the diameters determined from the XRD results in Sect. 3.2. Then by dividing A with reactor volume (${\text{V}}_{{{\text{react}}}}$), S is represented by Eq. (13):

13 $${\text{S}} = \left( {\frac{{\frac{{\text{m}}}{{{\text{V}}_{{{\text{react}}}} }}}}{{\uprho \cdot 4/3\uppi {\text{R}}^{3} }}} \right)4\uppi {\text{R}}^{2}$$

where the catalyst concentration (W) is given by W = m/V_react_.

#### Solution strategy

In order to find the solution to the model, the Eqs. (10) and (11) and Eq. (13) were coupled together and solved using an open source Python-based odeint() function which can be found from the Python-based scipy.integrate module. Odeint() is a built-in function in python that allows one to numerically solve ordinary differential equations. The model should be expressed in the form of Eqs. (14) and (15) in Python:

14 $$\mathop \text{x}\limits^{.} \left( \text{t} \right) = \text{f}\left( {\text{x}\left( \text{t} \right),\text{t},\text{p}} \right),\quad \text{t} \in \left[ {\text{t}_\text{o} ,\text{t}_\text{f} } \right]$$

15 $${\text{s}}{\text{.t}}{.}\quad \mathop {\text{x}}\limits^{.} \left( {{\text{t}}_{{\text{o}}} } \right) = {\overline{\text{x}}}_{{\text{o}}}$$

where x and p are differential variables and parameters, respectively, and f are the explicit ordinary differential equations. Equation (15) represents the initial conditions for the differential variables. A Python-based minimize() function from the scipy.optimize Python module was used to determine the parameters. Minimize() function contains a Sequential Least SQuares Programming (SLSQP) method which determines the non-linear model parameters by minimizing the specified normalized root mean squared error shown in Eq. (16):

16 $${\text{min }}\frac{{\sqrt {\frac{{\mathop \sum \nolimits_{{{\text{t}} = {\text{t}}_{{\text{o}}} }}^{{{\text{t}}_{{\text{f}}} }}\upvarepsilon _{{{{\rm Cr}}\left( {{\text{VI}}} \right)\left( {\text{t}} \right)}}^{2} }}{{{\text{z}}_{{{{\rm Cr}}\left( {{\text{VI}}} \right)}} }}} }}{{\overline{\upomega }_{{{{\rm Cr}}\left( {{\text{VI}}} \right)}} }}$$

where ${\text{z}}_{{{{\rm Cr}}\left( {{\text{VI}}} \right)}}$ is the number of data points for Cr(VI) reduction, $\overline{\upomega }_{{{{\rm Cr}}\left( {{\text{VI}}} \right)}}$ is the mean of the measured values of Cr(VI) concentration; and the residual of Cr(VI) ($\upvarepsilon _{{{{\rm Cr}}\left( {{\text{VI}}} \right)\left( {\text{t}} \right)}}$) is given by Eq. (17):

17 $$\upvarepsilon _{{{{\rm Cr}}\left( {{\text{VI}}} \right)\left( {\text{t}} \right)}} = {\text{Cr}}\left( {{\text{VI}}} \right)_{{{{\rm exp}}\left( {\text{t}} \right)}} - {\text{ Cr}}\left( {{\text{VI}}} \right)_{{{{\rm sim}}\left( {\text{t}} \right)}}$$

where ${\text{Cr}}\left( {{\text{VI}}} \right)_{{{{\rm exp}}\left( {\text{t}} \right)}}$ and ${\text{Cr}}\left( {{\text{VI}}} \right)_{{{{\rm sim}}\left( {\text{t}} \right)}}$ are the experimental and simulated results for Cr(VI) concentration respectively.

#### Parameter determination

**Figure 8:**
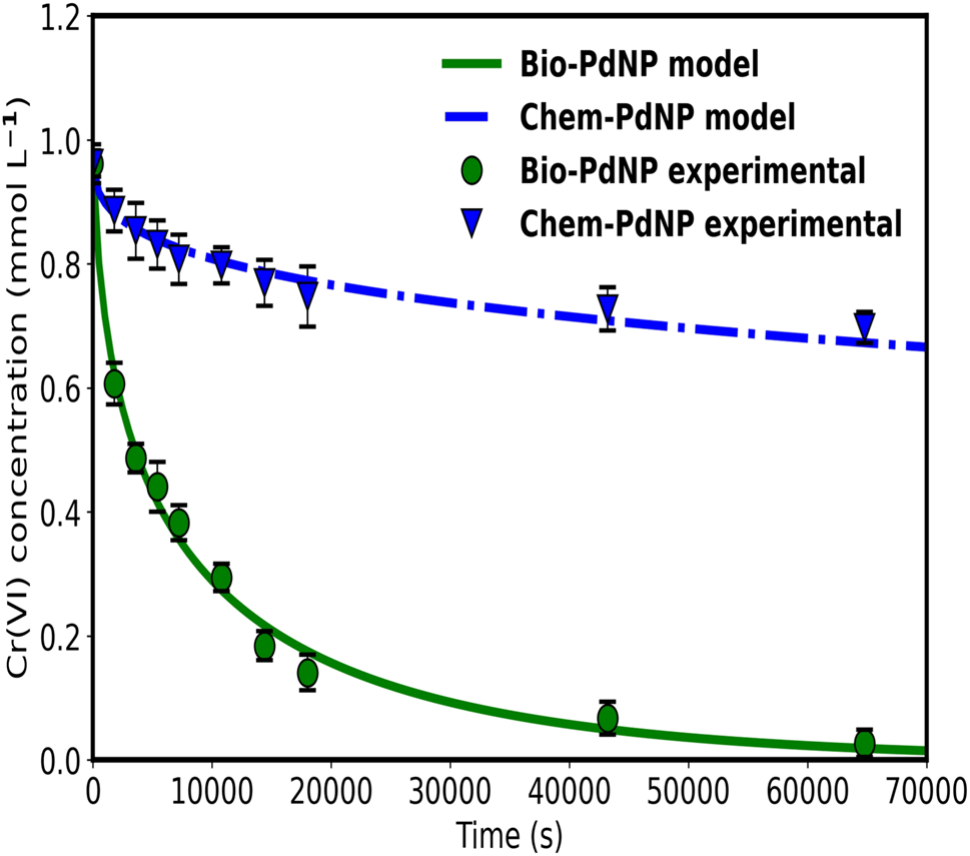
A comparison between predicted model values and experimental data for catalytic Cr(VI) reduction using Bio-PdNPs and Chem-PdNPs.

The parameters used in the kinetic analysis of catalytic Cr(VI) reduction were estimated, determined from literature and measured. Python-based minimize() function was used to determine the estimated parameters using the method SLSQP for non-linear parameter estimation problems from a Python module scipy.optimize and minimizing the objective function specified in Eq. (16). The parameters for Bio-PdNPs and Chem-PdNPs were estimated using the complete data in Fig. 8. Modeling results of catalytic Cr(VI) reduction are shown in Fig. 8.

The reason Bio-PdNPs performed better than Chem-PdNPs is extensively discussed in Sect. 3.3. Table 1 summarizes the model parameters values that were used for the catalytic Cr(VI) reduction model for Bio-PdNPs and Chem-PdNPs.

**Table 1:** Model parameter values for the catalytic reduction of Cr(VI).

Parameter	Units	Bio-PdNPs		Chem-PdNPs	
		Value	Ref	Value	Ref
k	mmol s^−1^ m^−2^	6.37	Estimated	3.83	Estimated
${\text{K}}_{{{{\rm Cr}}\left( {{\text{VI}}} \right)}}$	L mmol^−1^	3.11 × 10^−2^	Estimated	1.14 × 10^−2^	Estimated
${\text{K}}_{{{\text{HCOO}}^{ - } }}$	L mmol^−1^	1.12 × 10^−5^	Estimated	1.49 × 10^−4^	Estimated
${\text{K}}_{{{{\rm Cr}}\left( {{\text{III}}} \right)}}$	L mmol^−1^	2.76	Estimated	52.9	Estimated
ρ	kg m^−3^	11.8 × 10^3^	Constant	11.8 × 10^3^	Constant
R	m	10.9 × 10^−9^	Calculated	32.8 × 10^−9^	Calculated
V_react_	L	0.1	Measured	0.1	Measured
${\text{C}}_{{{\text{HCOO}}^{ - } ,{\text{o}}}}$	mmol L^−1^	111.1	Prepared	111.1	Prepared

As it can be seen from Table 1, the estimated parameters indicate that the rate constant ($\mathrm{k}$) of Bio-PdNPs is higher than that of Chem-PdNPs. In addition, the adsorption constant of Cr(VI) (${\mathrm{K}}_{\mathrm{Cr}(\mathrm{VI})}$) for Bio-PdNPs is higher than that of Chem-PdNPs, which indicates that Cr(VI) better adsorbs on Bio-PdNPs than Chem-PdNPs. It should be noted that the adsorption constant of Cr(III) (${\mathrm{K}}_{\mathrm{Cr}(\mathrm{III})}$) on Bio-PdNPs is lower than that of Chem-PdNPs. This indicates that Cr(III) better adsorbs on Chem-PdNPs than Bio-PdNPs.

Statistical analysis of the catalytic Cr(VI) reducing model using Bio-PdNPs and Chem-PdNPs was conducted in order to ascertain that the model was a good fit. The root mean squared error (RMSE) for each fit was calculated using Eq. (18):

18 $${\text{RMSE}}_{{{{\rm Cr}}\left( {{\text{VI}}} \right)}} = \sqrt {\frac{{\mathop \sum \nolimits_{{{{\rm i}} = 1}}^{{{\text{z}}_{{{{\rm Cr}}\left( {{\text{VI}}} \right)}} }} \left( {{\text{Cr}}\left( {{\text{VI}}} \right)_{{{{\rm exp}}\left( {\text{i}} \right)}} - {\text{ Cr}}\left( {{\text{VI}}} \right)_{{{{\rm sim}}\left( {\text{i}} \right)}} } \right)^{2} }}{{{\text{z}}_{{{{\rm Cr}}\left( {{\text{VI}}} \right)}} }}}$$

where ${\text{RMSE}}_{{{{\rm Cr}}\left( {{\text{VI}}} \right)}}$ is the RSME for Cr(VI). Then the RMSE was normalized using Eq. (19) to determine the normalized root mean squared error (NRMSE)^54^:

19 $${\text{NRMSE}}_{{{{\rm Cr}}\left( {{\text{VI}}} \right)}} = \frac{{\sqrt {\frac{{\mathop \sum \nolimits_{{{{\rm i}} = 1}}^{{{\text{z}}_{{{{\rm Cr}}\left( {{\text{VI}}} \right)}} }} \left( {{\text{Cr}}\left( {{\text{VI}}} \right)_{{{{\rm exp}}\left( {\text{i}} \right)}} - {\text{ Cr}}\left( {{\text{VI}}} \right)_{{{{\rm sim}}\left( {\text{i}} \right)}} } \right)^{2} }}{{{\text{z}}_{{{{\rm Cr}}\left( {{\text{VI}}} \right)}} }}} }}{{\overline{\upomega }_{{{{\rm Cr}}\left( {{\text{VI}}} \right)}} }}$$

where ${\text{NRMSE}}_{{{{\rm Cr}}\left( {{\text{VI}}} \right)}}$ is the NRMSE for Cr(VI). A NRMSE value of less than 0.15 indicates that a model is a good fit, and a value between 0.15 and 0.2 is still acceptable^55^. The results of RMSE and NRMSE for catalytic Cr(VI) reduction using Bio-PdNPs and Chem-PdNPs (Fig. 8) are summarized in Supplementary Table S2 online. As it can be seen in Table S2, both the NRSME values for catalytic Cr(VI) reduction using Bio-PdNPs and Chem-PdNPs achieved NRMSE ≤ 0.2. Therefore, the model fit for the experimental data in Fig. 8 was acceptable.

#### Model validation

**Figure 9:**
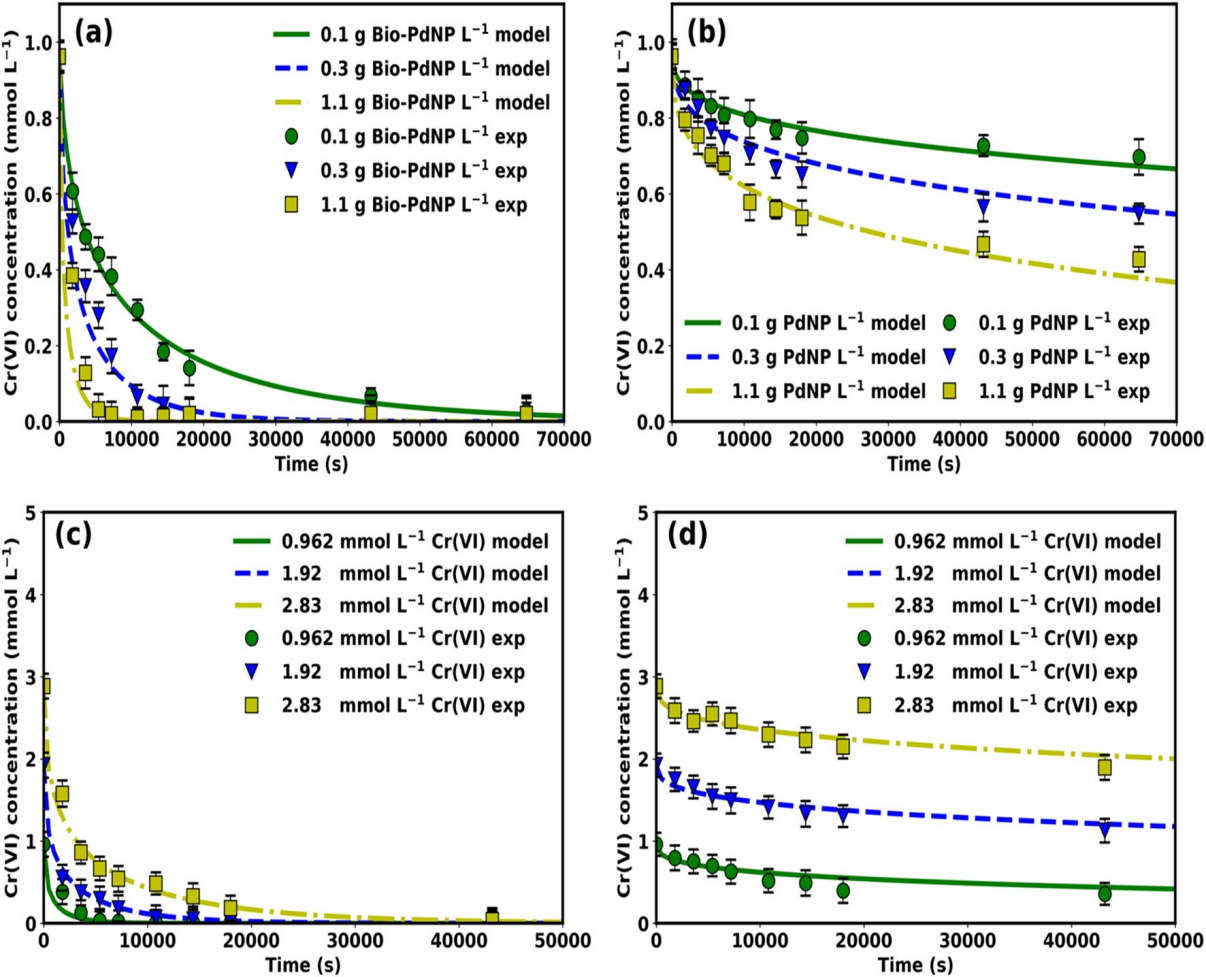
Model validation with experimental data at varying (**a**) catalyst concentration of Bio-PdNP at 0.962 mmol L^−1^ Cr(VI), (**b**) catalyst concentration of Chem-PdNP (represented as PdNP on the figure) at 0.962 mmol L^−1^ Cr(VI), (**c**) initial Cr(VI) concentration using 1.1 g Bio-PdNP L^−1^, (**d**) initial Cr(VI) concentration using 1.1 g Chem-PdNP L^−1^.

Model validation for the catalytic Cr(VI) reduction was conducted once all the parameters were specified. This was done by simulating the fully specified catalytic Cr(VI) reduction model and varying both the catalyst concentration of Bio-PdNPs and Chem-PdNPs. An open source Python-based odeint() function which can be found from the Python-based scipy.integrate module was used for the simulation and the results were compared to experimental data from Fig. 6. Figure 9a and b show the experimental and simulated results at varying catalyst concentrations of Bio-PdNPs and Chem-PdNPs, respectively. As it can be seen, there is a satisfactory agreement between the experimental and simulated values.

When using Bio-PdNPs, complete Cr(VI) reduction was achieved for all catalyst concentrations in 64800 s (18 h). Furthermore, Chem-PdNPs resulted in 27.4%, 43.1% and 55.5% Cr(VI) removal when using catalyst concentration of 0.1 g Chem-PdNP L^−1^, 0.3 g Chem-PdNP L^−1^, 1.1 g Chem-PdNP L^−1^, respectively (Fig. 9b). When increasing catalyst concentration of Bio-PdNPs and Chem-PdNPs, the rate of Cr(VI) reduction increased due to increased active sites as shown in Fig. 9a and b. In addition to the discussion in Sect. 3.3, the reason the Bio-PdNPs (Fig. 9a) performed better than Chem-PdNPs (Fig. 9b) was related to the rate constant and Cr(VI) adsorption constant for Bio-PdNPs being higher than that of Chem-PdNPs as shown in Table 1. Also, the product inhibition by Cr(III) was higher in Chem-PdNPs than Bio-PdNPs due to the adsorption constant of Cr(III) in Chem-PdNPs being higher than that of Bio-PdNPs as shown in Table 1.

For different catalyst concentrations, the total surface areas of Bio-PdNPs per reactor volume as per Eq. (13) were calculated as follows: S_0.1,bio_ = 23.32 m^2^ L^−1^, S_0.3,bio_ = 69.97 m^2^ L^−1^, S_1,1,bio_ = 256.57 m^2^ L^−1^. In addition, given that the rate constant of Bio-PdNPs given in Table 1 is k_bio_ = 6.37 mmol s^−1^ m^−2^, the product of the rate constant and total surface area of Bio-PdNPs per reactor volume were calculated as follows: k_bio_S_0.1,bio_ = 148.5 mmol s^−1^ L^−1^, k_bio_S_0.3,bio_ = 445.7 mmol s^−1^ L^−1^, k_bio_S_1.1,bio_ = 1634.4 mmol s^−1^ L^−1^. The total surface areas of Chem-PdNPs per reactor volume were calculated as follows: S_0.1,chem_ = 7.75 m^2^ L^−1^, S_0.3,chem_ = 23.25 m^2^ L^−1^, S_1,1,chem_ = 85.26 m^2^ L^−1^. In addition, given that the rate constant of Chem-PdNPs given in Table 1 is k_chem_ = 3.83 mmol s^−1^ m^−2^, the product of the rate constant and total surface area of Bio-PdNPs per reactor volume were calculated as follows: k_chem_S_0.1,chem_ = 29.7 mmol s^−1^ L^−1^, k_chem_S_0.3,chem_ = 89.0 mmol s^−1^ L^−1^, k_chem_S_1.1,chem_ = 326.5 mmol s^−1^ L^−1^. From the results, it can be seen that the product of the rate constant and total surface area per reactor volume of Bio-PdNPs was higher than Chem-PdNPs. This means that the Bio-PdNPs with the same catalyst concentration as compared to Chem-PdNPs had the highest catalytic activity which led to the highest performance.

An additional simulation on the effect of varying initial Cr(VI) concentration was conducted and compared to experimental results as shown in Fig. 9c and d for catalytic Cr(VI) reduction using Bio-PdNPs and Chem-PdNPs, respectively. It can also be seen that satisfactory agreement between the experimental and simulated values was achieved. When using Bio-PdNPs, complete Cr(VI) reduction was achieved for all initial Cr(VI) concentrations in 43200 s (12 h) as shown in Fig. 9c. In addition, Chem-PdNPs resulted in 62.8%, 41.3% and 34.2% Cr(VI) removal when using initial Cr(VI) concentration of 0.962 mmol L^−1^, 1.92 mmol L^−1^, 2.83 mmol L^−1^, respectively (Fig. 9d). The reason Chem-PdNPs did not result in complete Cr(VI) removal for all initial Cr(VI) concentrations and for the decrease in Cr(VI) removal rate with an increase in initial Cr(VI) concentration was due to the product inhibition since the adsorption constant of Cr(III) on Chem-PdNPs is high as shown in Table 1.

**Figure 10:**
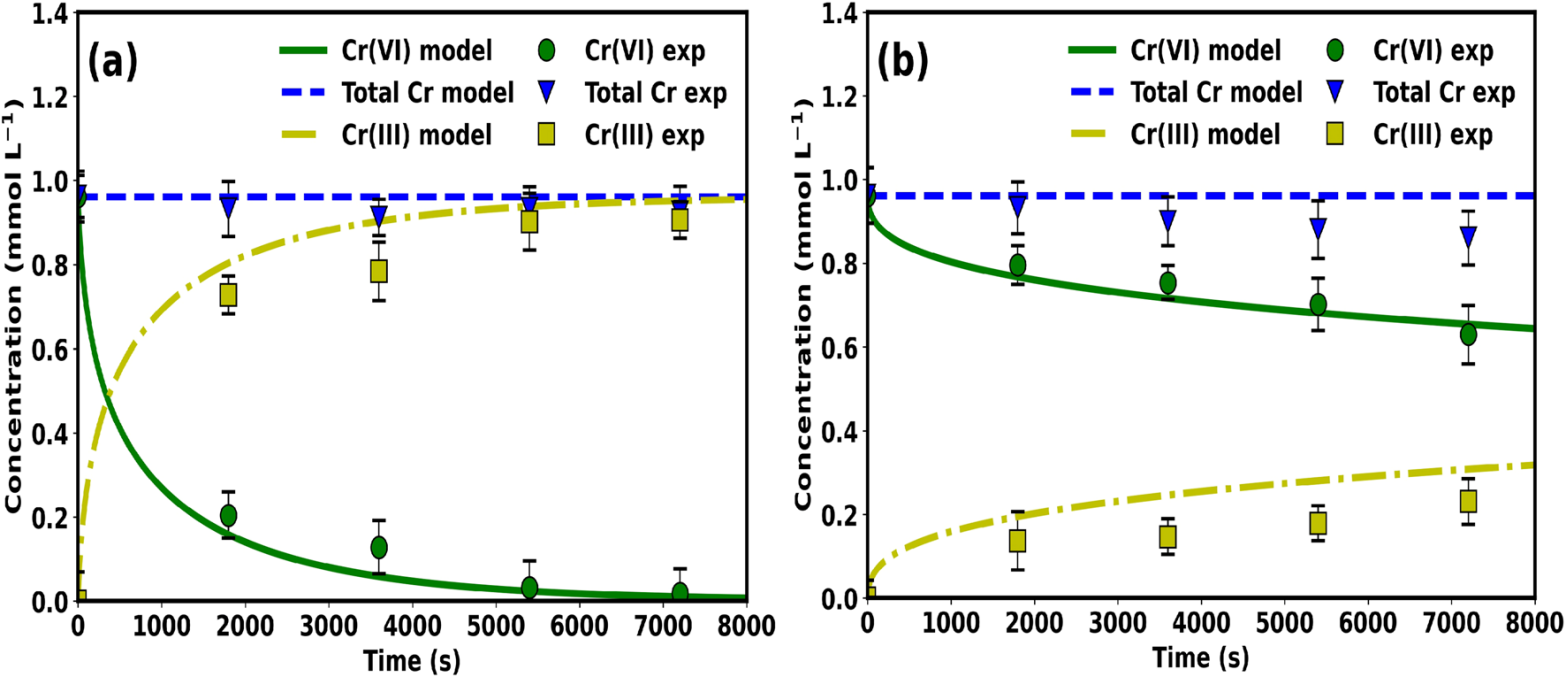
Reduction of Cr(VI) and product formation of Cr(III) when using (**a**) Bio-PdNPs and (**b**) Chem-PdNPs.

In order to also ascertain that the developed model has the capacity to predict the reduction product formed which is Cr(III) during the reduction of Cr(VI), an additional simulation was conducted. This was done by calculating Cr(III) concentration using Eq. (11) and determining the total Cr by the summation of Cr(III) and Cr(VI) concentrations. This was then compared with the results determined experimentally using the method detailed in Sect. 2.4. The results are shown in Fig. 10. As it can be seen in Fig. 10a, the prediction capacity for the total Cr and Cr(III) of the model was satisfactory when using Bio-PdNPs, however, in Fig. 10b there was lack of satisfactory agreement with experimental data when using Chem-PdNPs. This is attributed to the fact that the assumption made by the model that total Cr remains the same over time did not hold when using Chem-PdNPs. This means that Chem-PdNPs promoted the adsorption of Cr(III) as compared to Bio-PdNPs.

## Conclusions

In this study, successful synthesis and characterization of the Pd nanoparticles which were fabricated using the chemical and biological synthesis methods was demonstrated. During morphology analysis, Bio-PdNPs showed a roughened surface with many random and irregular arranged protrusions while the Chem-PdNPs showed a flower-shape with each petal having several smaller protrusions. Moreover, the EDS and XRD results confirmed that both synthesis methods resulted in elemental palladium. Due to the Bio-PdNPs being smaller in size and being highly dispersed as compared to Chem-PdNPs, this led to an improvement in the catalytic performance for Cr(VI) reduction when using Bio-PdNPs. Although both synthesis methods used in this study require less chemical agents which are less harmful and are cost effective, the Bio-PdNPs proved to be better since they can be synthesized within 24 h at a wide range of initial Pd(II) concentrations, low temperatures, and result in faster rates of Cr(VI) reduction. Successful kinetic modeling of the catalytic Cr(VI) reduction using the Langmuir–Hinshelwood mechanism was also achieved in this study. The Bio-PdNPs were shown to perform better than Chem-PdNPs due to the Cr(VI) adsorption constant for Bio-PdNPs being higher than that of Chem-PdNPs. Also, the product inhibition by Cr(III) was higher in Chem-PdNPs than Bio-PdNPs as shown by the high adsorption constant of Cr(III) in Chem-PdNPs.

## Supplementary Information

Supplementary Information.

## Abbreviations

*A*: Total Pd surface area (m^2^)
*C*: Concentration (mmol L^−^^1^)
*E*: Reduction potential (V)
*k*: Molar rate per unit square meter (mmol s^−^^1^ m^−^^2^)
*K*: Adsorption constant (L mmol^−^^1^)
*m*: Mass of Pd catalyst (kg)
*N*: Number of components (–)
*NRMSE*: Normalized root mean squared error (–)
*R*: Radius of the Pd nanoparticles (m)
*RMSE*: Root mean squared error (–)
*S*: Total surface area of the nanoparticles per reaction volume (m^2^ L^−^^1^)
*t*: Time (min)
*V*: Volume (L)
*W*: Catalyst concentration (kg L^−^^1^)
*z*: Number of data points (–)

## Species

[*Cr*(*VI*)]: Cr(VI) concentration (mmol L^−^^1^)
[*Cr*(*III*)]: Cr(III) concentration (mmol L^−^^1^)

## Greek

$$\varepsilon$$: Residual (–)
$\overline{\omega }$: Mean of the measured values
Ρ: Density of the metallic Pd (kg m^−^^3^)
$$\theta$$: Surface coverage (–)

## Subscripts

*an*: Anode
*Bio-PdNP*: Biologically synthesized palladium nanoparticle
*cat*: Cathode
*Cr*(*VI*): Hexavalent chromium
*Cr*(*III*): Trivalent chromium
*exp*: Experimental
*HCOO*^*−*^: Formate
*o*: Initial
*Chem-PdNP*: Chemically synthesized palladium nanoparticle
react: Reactor
*sim*: Simulation

## Superscripts

o: At standard conditions
