# Supplementary material for: Comparative analysis of biological versus chemical synthesis of palladium nanoparticles for catalysis of chromium (VI) reduction
Source: Sci Rep. 2021 Aug 17;11:16674. doi: 10.1038/s41598-021-96024-0 (PMC8371006; doi:10.1038/s41598-021-96024-0)
Supplement: Supplementary file 1 — Supplementary Information. [file 41598_2021_96024_MOESM1_ESM.pdf]

APPENDIX A: SUPPLEMENTARY DATA

Comparative analysis of biological versus chemical synthesis of palladium nanoparticles for catalysis of chromium (VI) reduction

Mpumelelo T. Matsena, Evans M. N. Chirwa.*

Water Utilisation and Environmental Engineering Division, Department of Chemical Engineering, University of Pretoria, Pretoria 0002, South Africa.

Corresponding Author

*Mpumelelo Thomas Matsena. Email: mpumelelo.matsena@gmail.com

1. Visual representation of the formed palladium nanoparticles.

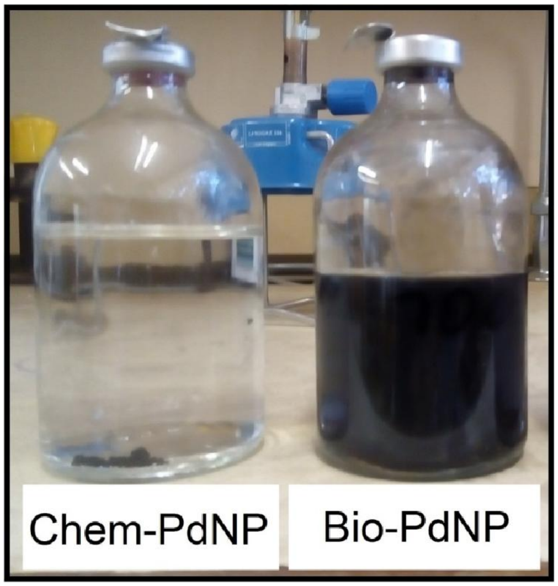

Fig. S1. Visual representation of the formed Chem-PdNPs and Bio-PdNPs.

2. Synthesis parameters of the palladium nanoparticles.

Table S1 Synthesis parameters for the characterized Chem-PdNPs and Bio-PdNPs.

Parameter	Chemical synthesis	Biological synthesis
Temperature	70 °C	30 °C
pH	6	6
Initial Pd(II) concentration	100 mg L^{-1}	100 mg L^{-1}
Experimental time	24 h	6 h
Carbon source	Sodium formate	Sodium formate

3. RMSE and NRMSE values for the catalytic Cr(VI) reduction using palladium nanoparticles.

Table S2 RMSE and NRMSE values for the catalytic Cr(VI) reduction using Bio-PdNPs and Chem-PdNPs.

Model fit	RMSE	NRMSE
Catalytic Cr(VI) reduction using Bio-PdNPs	0.022	0.061
Catalytic Cr(VI) reduction using Chem-PdNPs	0.015	0.019
